# We have never been ELSI researchers – there is no need for a post-ELSI shift

**DOI:** 10.1186/s40504-014-0009-4

**Published:** 2014-04-05

**Authors:** Bjørn Kåre Myskja, Rune Nydal, Anne Ingeborg Myhr

**Affiliations:** Department of Philosophy and Religious Studies, NTNU – Norwegian University of Science and Technology, NO-7491 Trondheim, Norway; Genøk – Centre of Biosafety, The Science Park, NO-9294 Tromsø, Norway

**Keywords:** ELSA, ELSI, Post-ELSI, Genomics, Nanotechnology, Emergent technologies, Interdisciplinary, Responsible research and innovation

## Abstract

This article criticizes recent suggestions that the current ELSI research field should accommodate a new direction towards a ‘post-ELSI’ agenda. Post-ELSI research seeks to avoid the modernist division of responsibility for technical and social issues said to characterize ELSI research. Collaboration and integration are consequently the key terms of post-ELSI strategies that are to distinguish it from ELSI strategies. We argue that this call for a new direction relies on an inadequate generalized analysis of ELSI research as modern that will affect the construal of post-ELSI strategies. We are concerned that the call for post-ELSI shift will exclude imaginative proposals and intellectual freedom by narrowing down the scope and methodologies of ELSI and thereby missing opportunities to play a critical and constructive normative role. Instead of framing current trends in ELSI research as a radical and progressive shift from ELSI to post-ELSI, we suggest an alternative story of expansion and diversification described in terms of a drift from ELSA 1 to ELSA 2, pertaining to acronyms in use in Europe. ELSI research has never been modern. It has been experimenting from the very start on ways to mesh the works of humanist, social and natural scientist in order to bridge and build alignments of emerging scientific and societal goals and matters of concern. The development from ELSA 1 to ELSA 2 expands in our account the range of intellectual and methodological capacities of analysis and engagement of complex and dynamic science-society relationships. We present three areas of ELSA expertise to illustrate that the expertise within the field builds on scholarly achievements within the humanities, social sciences as well as the natural sciences. The plurality of disciplinary background of ELSA researchers represents a valuable diversity that enables mutual criticism and formulations of complementary approaches that together constitute a viable ELSA field.

## From ELSI to post-ELSI

In a series of seminars funded by the British ECRS, a group of researchers explored possibilities of building a “‘post-ELSI’^a^ interdisciplinary research agenda” (Seminar 5: 2012). This work resulted in what was nicknamed a post-ELSI manifesto (Balmer et al. 2012). The manifesto calls for a new role for social researchers in interdisciplinary technology development projects replacing the one allegedly often given them as “joyless, humourless ‘nay sayers’” in charge with “determining the ethical or social consequences of technical developments”. The roles of social scientists in such projects “are often limited to narrow, prescriptive positions that have been entrenched through funding arrangements, disciplinary and institutional boundaries, governance regimes and local politics” (Ibid.). Such narrow societal imagination of the role of the social scientist, according to the manifesto, follows from a normative division of professional labour between natural and social scientists that is conceptually and institutionally entrenched in our culture. Instead of separating the responsibilities of natural and social scientists, the manifesto argues that they should be seen as crossing over each other’s domains:

*Instead of dividing up responsibility for the technical and social along lines of natural and social sciences, we see them as deeply entwined. This means both natural and social scientists can work together to produce a critical and human understanding of how design, development and application of new technologies are accomplished. (Balmer et al.*^2012^*)*

Some important theoretical presuppositions appear to be at work in the manifesto. The established division of labour described in the manifesto, mirrors a flawed linear understanding of the relationships between technological and social activities. These relationships should not be imagined as linear, although we seem to spontaneously do so, but rather as nonlinear with complex feedback loops, simply because such imaginations actually do capture better how these activities interact. A prescriptive role of the social scientists follows from this philosophy of science; they should not work in parallel, alongside or after the natural scientist – they should seek true collaboration. ‘ELSI’ is associated with the former strategy while ‘post-ELSI’ is connected to the latter. The positive message of the manifesto consequently consists in seven principles for “experimental collaborations” between social and natural scientists.

“Post-ELSI” is an interesting grammatical construction like several similar constructions, such as post-modernism, post-feminism and post-humanism. The positioning of the “post”-movement is usually done through a distancing from some assumed key aspects of the practice or phenomenon that the proponents of the novelty seek to overcome. The same is the case with post-ELSI: the call is based on the perceived shortcomings of ELSI research. But raising an alternative or an improvement to ELSI research, presupposes that there exists a form of research that is rightfully called ELSI – just as the relevance of a post-modern challenge presupposed that there was something rightfully called modern (Latour 1993, 10). Whatever activity “post-ELSI” is to prescribe will be marked by the normative diagnosis of the ELSI research it presupposes. Inspired by Latour’s analysis of the need to rethink modernity in our attempt to replace it we ask the question: What if we never have been ELSI researchers? Attention would be drawn to how post-ELSI envisions itself as continuing or fulfilling the task ELSI was to achieve. Normative discussions of urgent issues of science and technology developments could then play a more formative role in shaping adequate ELSI strategies.

The manifesto calls for a rethinking of the role of social scientists in times where collaboration “between scientists, engineers and social scientist” has become “commonplace” (Balmer et al. 2012). The manifesto does not give much hint to why calls for collaboration have become commonplace, but such calls for collaboration seem to be taken as expressions of a genuine need. We are, as seems to be assumed, in the midst of transitions calling for a shift from ELSI to post-ELSI research agendas. According to the manifesto, we do not quite know how to carry out these transitions, hence the need for “experiments of collaboration”.

We realize the dangers of relying too heavily on the wording in manifestos and workshop descriptions, but we have grounds for holding that the proponents of the manifesto wish to distance themselves from the dominant ways of conducting ELSI research. They express this clearly in an invitation to one of the previous workshops in the series, where they claim the problem with ELSI research is to be found in “the timeliness of social science interventions and the implied distinction between scientific research on the one hand and social implications on the other” (Seminar 3: 2011). The “timeliness” of social science intervention is here made with reference to several ELSI research programs’ focus on “implications of new technologies”. It is difficult not to read the call for ‘post-ELSI’ as carrying a normative diagnosis of ‘ELSI’ research activity. This normative diagnosis appears to have a theoretical and a practical component.

Theoretically, a post-ELSI strategy is assumed to be framed by what is seen as a more viable philosophy of science. An ELSI research strategy, as it appears to us, is more specifically understood as tokens of being bound by what Latour (1993; chapter 2) called the modern constitution prescribing a sharp division of labour between natural scientist on the one hand and humanist and social scientists on the other hand. The normative work within the modern constitution is in this analysis conditioned by what Latour called a "work of purification": The realm of fact and value, nature and society should carefully be purified, separated and subsequently treated in different realms, defining among other things the work descriptions of the humanist and social scientist. The modernist normative commitments guiding the practitioners’ respective work descriptions facilitate in turn science and technology development by rendering its dynamics invisible. Scientific and technological development is not in practice bound by the modern constitution. On the contrary, it succeeds because it constantly creates complex sociotechnical associations through what Latour described as “work of translation”. This dynamics of socio-scientific developments has not commanded our attention precisely because of our common focus on the work of purification: everybody should see that this is done properly and not interfere in each other’s domain and should first of all be deeply immersed in one’s own work domain.

Our concern here is not Latour’s analysis of modernity, but the question of how well ELSI fits into a picture of an activity that is bound by the modern constitution, while its replacement, the post-ELSI strategies are not. This is where what could be called practical assumptions of ELSI research becomes important; the assumption that ELSI strategies actually fails to instigate normative work. If a call for a post-ELSI strategy is to make sense, ELSI must be replaced rather than adjusted and complemented. The post-ELSI analysis stands in danger of denying the complexity it assumes, which may result in a narrowing down of the range of ELSI strategies instead of opening them up.

A closer look at ELSI research as it has been carried out will indicate to what extent the post-ELSI criticism is based on an adequate understanding of the ELSI field, and simultaneously question whether there are grounds for turning towards post-ELSI strategies. We will argue that post-ELSI calls do not rest on an adequate description of ELSI research. ELSI research has not generally delivered low quality or irrelevant normative analysis. We are not convinced that urgent issues concerning technology development are better targeted through what is described as post-ELSI strategies. In this article, we challenge the basic assumptions of the post-ELSI call through an analysis of ELSI research from its early start in the Human Genome Project. We question the understanding of ELSI research implied by the call for post-ELSI strategies by offering an alternative analysis. We hold that the strategies named post-ELSI in the manifesto has already been recognized as ELSI strategies worth pursuing, developed as an essential element in the self-reflexive, on-going discussions within the ELSI field itself concerning how to carry out this research. We agree it makes empirically sense to speak of a shift in ELSI strategies, but there is no need to build a new research agenda since this shift has taken place in what we describe as a transition from ELSA 1 to ELSA 2, to use the acronyms from the EU and Norwegian contexts.

Our claim is that ELSA 2 should be understood as an extension of ELSA 1, consisting of an overlapping conglomerate of methods, topics and theories being developed through more than two decades of ELSA research programs. As such, there is nothing to overcome, no need for a post-ELSI agenda, as there has never been a specific ELSI strategy as implied in the call for post-ELSI strategies. On the contrary, the rhetoric of the ELSI – post-ELSI dichotomy stands in danger of strangling ELSI activities, turning them into a distinct, more or less unified field defined within specific disciplinary capacities in ways that can weaken the normative vigour of ELSI.

## The criticism of ELSI

ELSI was originally an acronym for the Ethical, Legal and Social Implications Research Program of the Human Genome Project between 1990 and 2003. “The planners of the Human Genome Project (HGP)”, as stated in the web page of the US National Human Genome Research Institute, established the ELSI Program because they “recognized that the information gained from mapping and sequencing the human genome would have profound implications for individuals, families and society.” (NHGRI 2012)

The ELSI program of the HGP was a scientific pioneer program as it also allocated research funding for social scientists; it positioned “bioethics inside the beltway” as Eric Meslin and his colleagues put it in a title of an article. The goals of this ELSI program were

*(1) to develop a program to help understand the ethical, legal, and social implications of the human genome project, and (2) to identify and define the major issues of concern and develop policy options to address them. (Meslin et al.*^1997^*;292)*

And during the first years ELSI research came to be focused on four priority areas:

*(1) privacy and fairness in the use and interpretation of genetic information, (2) clinical integration of new genetic technologies, (3) issues surrounding genetics research, and (4) public and professional education. (Ibid.: 294)*

What then, is fundamentally wrong with the ELSI program, as it is carried it out within the HGP? It seems to be a responsible funding strategy to supplement innovative and potentially controversial genomics research with research that aims at anticipating implications such increased knowledge would have in terms of identifying “major issues of concern”. The general assumption was, and is, that such knowledge could have profound impacts in our societies, individually and collectively. Accordingly, the quality of the research within the ELSI program should therefore concern questions of whether one really manages to identify and explore the issues that can have social, ethical and legal impacts. The question of identifying the “major issues of concern” is also connected to the post-ELSI claim of ELSI research not being on the right track in terms of targeting the right places, places where our common future is shaped.

We have, however, difficulties in finding literature that call for post-ELSI strategies based in detailed moral and political discussions demonstrating the fallacies of previous or present ELSI research or literature that engage in discussion on what the real major issues of concerns should be. The issue here is not one of establishing a position from where one may make claims regarding what the crucial concerns are. It concerns the need for engagement in substantial discussions of what decisions should de facto be made at the policy level, what decisions should be made elsewhere, and how studies of the dynamics of science and technology can and should improve these decisions. Without such analysis it is difficult to find valid reasons for replacing current ELSI strategies as well as to identify what strategies that actually are criticized.

One main post-ELSI criticism of the whole ELSI research program and the ELSI field at large is that it has been too oriented towards the consequences of the research and development process. Thereby the politics of the research process has been concealed in ways that in fact serve the interests of the technology proponents, as well as creating a comfortable professional zone for the ELSI researcher (see for instance Balmer and Bulpin 2013). In many cases, these may be reasonable suspicions and important issues to raise with respect to the quality of ELSI research programs. When the institution funding ethics research has particular interests, for example to promote biotechnology, as was the case with the HGP, asking questions and finding answers that are compatible with the interests of the funding agency might be tempting. After all, during the first 13 years of the HGP, they allocated 125 million ELSI dollars to ELSI research, having a considerable influence on the field of bioethics in particular and the field of applied ethics in general (Powledge 2003). The implicit accusation is that ELSI researchers go where the money takes them, echoed in Art Caplan’s calling the ELSI program “the Full Employment Act for bioethicists” (Today in Science Quotes 2013). Langdon Winner, testifying to the US congress, sharpened the criticism of this research: “there is a tendency for those who conduct research about the ethical dimensions of emerging technology to gravitate towards the more comfortable, even trivial questions involved, avoiding issues that might become a focus of conflict”. He added that people in bioethics “rarely say ‘no’” (Winner 2004)^b^.

It is difficult to evaluate claims such as Winner’s due to their low precision and lack of documentation. After all, the context of this statement is a testimony, not an academic publication. Still, we would expect Winner or those who refer to his testimony to be able to back up such claims by some form of documentation. Assessing the relevance of Winner’s and others’ claims concerning the character of ELSI research, should in addition be framed as a normative discussion of what the real and important research questions and issues are, or what a legitimate procedure would be to settle such issues.

We can interpret Winners statement in a weak, trivial sense, or in a strong, controversial one. It is sufficient that a few of those doing projects for the ELSI program to “gravitate towards the more comfortable” for it to be true in the weak sense. It is very likely that at least some ELSI projects were of this “yea-saying” kind, but that is of little value as critique of the program as such. Assuming that there also was significant and influential research that was grappling with the difficult and conflict-filled issues, we should not be too concerned that some did poor research. If this is all he intends to say, his statement is of little interest and value.

The potential thrust of Winner’s statement lies in the stronger claim that what characterizes ELSI research *in general* is this tendency towards the comfortable and affirmative. It is unlikely that the funding sources determine research results by necessity, so we must understand Winner’s claim as an empirical statement about ELSI research. Winner is not the only one to make such claims, and influential researchers have repeatedly expressed similar judgments with the same lack of empirical support (Kitcher 2001, Barben et al. 2008). It falls easy to read this as research politics promoting certain interests rather than as scientifically valuable analyses.

An ELSI approach geared towards consequences of research and development processes may be accused of serving the interest of technology proponents. In addition, consequence-oriented research stands in danger of ‘coming too late’, and thereby masking the immanent ethics of science inherent in the messy process where courses of science, society and politics are formed.

Two influential approaches, the Dutch Constructive Technology Assessment (CTA) (Rip et al. 1995) and the American Real Time Technology Assessment (RTTA) (Guston and Sarewitz 2002) explore ways of engaging researchers, industry partners, government officials and stakeholders in the research process challenging ELSI researchers to play a productive and constructive role. These approaches are indeed innovative ELSI approaches that may potentially be more challenging for the research and development program it is to investigate.

In a couple of passages, Paul Rabinow and Gaymon Bennett (2009, 106; 2012;15) make a strong and disturbing claim, that the ELSI program of the HGP were deliberately designed not to interfere with the research program itself. “These programs were constituted according to the terms of a political agreement among the Human Genome Project funders that ELSI would be supported on condition that it operated downstream of the science and technology, and should concern itself primarily with framing *social consequences*”. The ELSI programs of the HGP, as portrayed by Rabinow and Bennett then, could systematically carry blind spots, not only failing to support research possibly able to address important issues, but also function as a door-keeper ensuring them not being raised at all.

One can find support for these claims in Robert Cook-Deegan’s (1995; 231 f) account of the history of the HGP as Cook-Deegan ties the growing political support for ELSI to specific worries of social consequences like increased prenatal diagnosis and abortion. The HGP would be put in jeopardy without an explicit attention to such ethical issues, which in turn came to mark the ELSI program, as Cook-Deegan put it “Where the cash went, Ethics followed (ibid: 241)”. What this implies for the identity of ELSI researchers is, however, not obvious. Other participants of the ELSI program like Michael Yesley (2008;4) are critical to how the organisation of ELSI research tended to suppress issues like priority, genetic reductionism and biological warfare. Daniel Callahan (1996;3) seem to suggest that the problem of co-optation was something the community came to experience as a problem following their success of convincing scientist to consider them as “allies and not opponents [..] We became insiders by default, without ever resolving in any full way the question of whether those who pursue bioethics should be insiders or outsiders”. Public statements from key persons in the first ELSI program, as we will see below, do not support a picture of ELSI as deliberately on the ‘outside’ and ‘downstream’, either. Rather than a story of deliberate non-collaborative strategy, the story could be told as a story of the pioneers of collaboration.

Rabinow and Bennett did get funding, not to do ELSI connected to HGP, but to include a “social implications” component in a synthetic biology initiative. They describe a process where the design of such a component was open for negation from the start. The meaning of “social implications” was neither clear to officials from the National Science Foundation nor principal scientist and engineers that were to set up and coordinate the Synthetic Biology Engineering Research Center (SynBERC). Translating “social implications” into a mode where Rabinow and Bennett would be seen as active collaborative partners in the creation of the Center did not seem to be controversial either. On the contrary, Rabinow were, somewhat to his own surprise, invited to become an active participant. In this context, they saw this as a novel challenge they understood as putting “into practice a form of ‘post-ELSI’ program” (Rabinow and Bennett 2012;18). The notion of post-ELSI seems in this context first of all to be connected to issues of how to become active participants. Rainbow had already contributed to the development of synthetic biology as an “anthropological observer”, he now were to contribute as an anthropological participant.

As far as we can see, Rabinow and Bennet are the first to use the term “post-ELSI” approach, carrying a call for a “post-ELSI” ethics; a shift that they argue is already taking place but requires an experimental attitude. One of these post-ELSI approaches is their own “Human Practices” (HP) approach (Rabinow and Bennett 2009; 100). Collaborative relationships between social scientists and natural scientists are a key concept of HP pinpointing a professional challenge for the practitioners, as they are to become participatory rather than observational anthropologists.

The novel challenges of post-ELSI approaches include questioning established division of labour in ways that involve a rearticulation of the practitioners’ own professional identities (Rabinow and Bennet 2007). However, this is not significantly different from the challenge experienced by ethicists some decades earlier. In his classic article ‘How medicine saved the life of ethics’, Stephen Toulmin (1982) described the professional challenges ethicist experienced in facing novel and practical ethical dilemmas in medicine, often induced by the introduction of new technologies. The ethicists’ approaches had to be reinvented in light of the challenge of providing productive input to professionals in the field faced with ethical issues that would not go away.

Ethicists working in the ELSI program, as we see it, had already faced a similar professional challenge to the one Rainbow and Bennett describe within their field, and the ELSI program was one important venue for them to investigate, develop and adjust the approach sometimes referred to as Applied Ethics. The ELSI programs were so to speak a post-ELSI program from the very start, investigating new modes of engaging various forms of expertise, where ethicists where challenged to take the step from observational to a participatory ethicist.

We do not question the core post-ELSI emphasis on the need to experiment on new forms of collaboration. What we are concerned about is the further work the notion of collaboration does when the post-ELSI term appear in generalized calls for a shift as in the post-ELSI manifesto. Crucial questions are questions like; what is it actually that needs to be brought together (or integrated) through collaboration (like knowledge, research activity, institutional considerations, moral commitments)? How is it presumed that collaboration can contribute to increase the quality of the work (of the respective discipline, the interdisciplinary research activity and the policy agencies)? Where is collaboration to take place, is it in the lab, in the committees or though efforts of maintaining diversity and mutual criticism? What are the respective methodological challenges of the various disciplines involved (that may vary from disciplines within the humanities, social sciences and the natural sciences)?

The notion of Post-ELSI stands in danger of de-historicize the core notions of collaboration and integration its proponents seek to draw attention to, and thereby threatens to bracket off lessons of earlier experiments while simultaneously narrow down the open and complex set of research agendas they seek to establish. ELSI research programs play an important role in defining what a post-ELSI shift is about as it epitomises what *not* to do. As ELSI programs are presented as the paradigmatic case of non-collaborative activity to be transgressed, they simultaneously constrain the imagination of why, how and where collaborative activity is liberating, who it is liberating for and what the standards of success are. ‘Collaboration’, we maintain, along with ‘integration’, have always been key terms of ELSI research although not always equally well articulated as core concepts. The challenge these concepts expresses should not be trivialized by, for instance, equating the term with being what can be termed ‘active’ or ‘involved’ (sociological) research partner at the laboratory floor.

The initial ELSI program was a novel construal it has taken time to digest and develop. James Watson surprised everybody, presumably himself included, when he impulsively announced the creation of the ELSI program of the HGP at a press conference in 1988 (Juengst 1996; 63). Although this has widely been regarded as a strategic move to forestall critique and secure support for the HGP, Watson himself has said that he proposed the ELSI program because there are genuine worries that needed to be addressed:

*“Good science affects its social context [… ]. Science, in turn, is constantly affected by the professional norms, social politics and public perceptions that frame it. […] Doing the Genome Project in the real world means thinking about these outcomes from the start, so that science and society can pull together to optimize the benefits of this new knowledge for human welfare and opportunity.” (Watson and Juengst 1992, cited in Juengst 1996: 67)*

There is, as we see, a rhetoric indicating sensitivity to the social context and the interplay between science and society at this early stage of the ELSI project. Juengst points out how the scientists involved in the HGP had background from the early recombinant DNA research with focus on biohazards as expressed in the Asilomar moratorium. Therefore, at least for some of them, “participating reflectively in public discussions of their work and incorporating the research is accepted as a natural and necessary part of doing science” (ibid. 68).

Thus, the notion of post-ELSI draws attention to recurrent topics discussed in the ELSI program from the start. Juengst partly foreshadows the criticism of ELSI programs serving the interest of the technology proponents by pointing out that the aim of social responsible genomics research rules out ELSI research questioning the HGP as such (ibid. 69), but as he adds, no one is prevented to do that from the outside of the project funding. Moreover, the ELSI program actually contributed to mediate and engage with such criticism. In an early ELSI publication, *The code of codes* (Kevles and Hood 1993), three such “outsiders” (Ruth Cowan, Evelyn Fox Keller, and Dorothy Nelkin) were included in the book in ways that “captures both promotional and critical perspectives on the genome project” as Susan Lindee (1994) put it in her review of early ELSI literature. Lindee also shares the worry that basic upstream questions of priorities of the HGP where officially outside ELSI territory. If the project cannot be expected to evaluate itself, it drifts towards enforcing the division between “’science’ and ‘implications’ of that science”. Kathi E. Hanna (1995), in an evaluation report of an ELSI program, likewise criticised the program for being “overly academic”. The program would need to diversify if one should not miss this excellent opportunity to make a difference for a scientific project like the HGP.

The ELSI program of the HGP became the paradigm for similar programs in Canada and Europe, but they have taken on different forms relating critically to the notion of “implications”. In the EU and Norway, “aspects” replaced “implications”, leading to the acronym ELSA, signalling more than a mere shift of name. A similar shift happened in the US and within the HGP itself, where “issues” replaced “implications”, thus keeping the acronym while changing the meaning. This shift is clearly indicated in some of the official statements, contradicting a simple picture of ELSI research only concerned with regulation of downstream consequences: The research should “guide the conduct of genetic research and the development of related health professional and public policies” (Collins et al. 1998).

Several of the early ELSI projects were highly critical of the presuppositions and goals driving the HGP as Jungest (1995: 75–76) points out. In a later review of ELSI literature Audra Wolfe (2001) makes a similar point, drawing attention to a number of works (such as Lilly Kay’s *Who wrote the book of life?*) that critically undermine the core legitimizing metaphors of the HGP.

The call for a post-ELSI shift, to summarize, seems to rely on three main lines of arguments of why ELSI research programs fails as normative programs:

- ELSI research is geared towards researching ‘consequences’ of research in ways that make it blind for the politics of research and draw attention away from important matters of concern.
- They lead to comfortable and affirmative research. Perhaps even worse, the research may sidetrack into nurturing internal discussions among ethicists that do not hook up with the world beyond the disciplinary boundaries it creates for itself.
- There is an unfortunate normative division of labour in force between ELSI researchers and the natural scientists. A radical shift of research strategy is needed; one that runs under the heading of integration and collaboration.

We suggest an alternative story understanding ELSI programs as having had a collaborative agenda from the very start. Through critically scrutiny of the productivity of the way the program has been organized, it has been able to improve itself by diversifying and expanding its research strategies. The post-ELSI approaches rests on a caricature of ELSI as non-collaborative, uncritical and non-reflexive, which in turn makes it possible to present an alternative to ELSI research in positive terms of collaboration and integration. More important, the post-ELSI call rests on an unwarranted normative diagnosis of ELSI research, as it is not supported by normative substantial analysis.

Our worry concerns the way the post-ELSI story seems to become a mainstream story that, in effect, restricts the scope of ELSI. Examples of such restrictions include recent ELSA calls from the Research Council of Norway’s dedicated ELSA program and programs funding large-scale bio- and nanotechnology research (ELSA 2 2008). These calls, clearly inspired by a post-ELSI agenda, excluded a range of imaginative proposals and analytical tools by asking exclusively for “experiments of collaboration” (such as mandating ELSA work packages in every scientific research projects)^c^. The early proposals for EU’s Horizon 2020 displayed a similar tendency, replacing general ELSA-programs within the funding structures with integration of ELSA research within large interdisciplinary science projects. In the European Framework Programmes, ELSA-like activities, especially related to bioethics, have been a component since the Second Framework Programme (1987–1991). A shift in focus was presented with the EU’s Science and Society Action Plan from 2002, which emphasizes the importance and the role of new technologies for developing responsible science. At present ELSA has become distributed – consisting of a series of work-packages integrated into scientific research projects (in order to promote collaboration) (Stegmaier 2009).

Thus, we hold that the call for post-ELSI experiments is based on a misleading analysis of two decades of ELSI research, in ways that narrow rather than expand ELSI capacities. Furthermore, a one-sided focus on post-ELSI collaborative approaches stands in danger of covering up the issues related to different dimensions of power in ELSA research. Dealing with these problems requires that the integrated collaboration be supplemented by independent, external scrutiny. In the following pages, we sketch an alternative story drawing on a report commissioned by the Norwegian ELSA research programme (Nydal et al. 2011).

## From ELSA 1 to ELSA 2

HGP’s ELSI program was not only imitated in other countries, but also in other fields, like nanotechnology and synthetic biology, the research context of the formulation of the post-ELSI manifesto. The context of ELSI matters as new arenas provide valuable opportunities to revisit questions of how to model an ELSI component of such priority areas. The wider context of ELSI of emerging technologies, we suggest, differs in three ways with respect to that of early genomics. These differences have in turn implied a set of novel possibilities and challenges for ELSA scholars. In the process of dealing with these challenges, in part by exploring novel possibilities, ELSA has shifted character in ways we prefer to describe as a development from ELSA 1 to ELSA 2, rather than as a shift from ELSI to Post-ELSI.

First, the HGP comprised early phases of what has widely come to be recognized as contemporary trends towards a more context driven research with focus on the context of application (Gibbons et al. 1994, Nowotny et al. 2001 and for similar analysis see Ziman 2002, Funtowicz and Ravetz 1993). Genomics and nanotechnology, in contrast to earlier icons for the frontiers of science like high-energy physics, seek socio-economic measures of legitimacy in addition to basic understanding. Novel arenas for ELSA, such as nanotechnology, appeared in times where these analyses have increasingly become assimilated, reflected among other things in science policy documents accompanying nanotechnology initiatives worldwide (e.g. European Commission 2004, The Royal Society & Royal Academy of Engineering 2004, Nordmann 2004). These documents call for incorporation of ethical and social considerations in research and developmental processes (“upstream”). Having an ELSA component of scientific priority areas does no longer seem to be an oddity, nor particularly controversial in ways that create new opportunities of collaboration.

Second, nanotechnology appears against a learning process of early attempts to integrate ethical and societal consideration at an early stage of the development process, among which we find the first ELSA initiatives (see e.g. ELSA 1 2001). In this respect, nanotechnology research encounters an already established field, as it was possible to imagine outcomes of different ELSA approaches. The shift of empirical setting as well as accompanying scholarly networks adds to the possibility of rethinking goals and approaches of ELSA studies, and thus, for what kind of knowledge and impact this research should aim.

Third, the technological possibilities emerging at the initiation of the HGP were already disclosing some clear ethical problems and challenges. The nanotechnology ELSA calls, however, came before anybody clearly knew the ethical and social issues that may arise due to the development of these technologies (Nordmann 2004). Thus, extending the idea of having an ELSA of nanotechnology focused the challenge of how to scrutinize technologies ethically as they evolve. This focus contributed to the enrolment of novel ELSA type of studies sensitive to the temporal dimensions of technology development, such as Real-time technology assessments.

In particular, ELSA-studies of nanotechnologies are well suited for the constructivist STS (Science and Technology Studies) field. The lessons and methods of STS are attuned to the study of processes where technology is being constructed (as opposed to a conception of science as ready-made). The ability to trace and understand the dynamics of developmental processes is one of the main strengths of constructivist approaches. As we do not know the socio-ethical challenges that nanotechnology will give rise to since we do not know the potential of the technology, it is a reasonable strategy to pay close attention to the how nanotechnology emerges in a range of different cases as they evolve in practice.

These three elements, we suggest, make it reasonable to speak of a shift from the initial ELSA approach associated with the HGP, to the ELSA of novel arenas such as nanotechnology, what we refer to as ELSA 1 and ELSA 2. It is important to notice, in conjunction with this analysis, that although several challenges within these kinds of research collaborations are the same across different technology areas, many are specific. For example, the ELSA of synthetic biology does not face the same challenges as ELSA of population-based biobanks. This is another reason for being suspicious for generalizing claims of the kind witnessed in post-ELSI calls.

The transition from ELSA 1 to ELSA 2 is not one of a clean break calling for new researchers and other disciplines. Research groups active in the early days are still part of the ELSA research community, although others have joined them, as part of the transition. It would be wrong to claim that ELSA 2 represents a new research paradigm, as the main elements of the transition is already present in the HGP phase, including integrated research and fostering improved communication between science and society. At this point, we will return to Latour’s analysis of modernity in order to contextualize our criticism of the post-ELSI initiatives, especially focusing on the need for interdisciplinary.

### Conservation and development of diversity within ELSI/ ELSA research

There are some clear similarities between the post-ELSI and the post-modernity rhetoric. In both cases, the dual work of modernity is not taken into consideration. The work of translation does not capture our attention due to the way we purify phenomena through the organisation of our investigations. The concepts of ELSI like modernity are themselves constituted as purified objects in order to create a clear and distinct entity that has outlived its day and needs an alternative. Such construal hide the ELSI activity of maintaining and transforming itself through constantly violating the very distinctions that is to define ELSI. However, in order for such entities to exist, the details of the messy reality must largely remain not investigated, maintained by vague and loose claims of the nature of the activity. Just like modernity is a concept that belies the phenomenon that it refers to, ELSI is not captured by simple descriptions such as ‘downstream’, ‘consequence-oriented’, ‘affirmative’, ‘comfortable’ and ‘under a clear labour division with technoscience’.

In order to move downstream, you must interact in some ways with the upstream research, and the moment you become merely affirmative you lose credibility and the natural scientists will not find it worthwhile listening. As we saw above, many of the early HGP researchers belonged to the Asilomar generation and were truly worried about the power within genetic engineering. Thus, if ELSI research had fitted the rhetoric of its critics, it could not survive to be superseded by post-ELSI. However, as the truth is messy and vague, it is not found in the negation of the post-ELSI rhetoric, either. ELSI did not contain the post-ELSI strategies from the outset. These strategies have gradually emerged, as part of the research field due to internal debate and external criticism, and the result is the gradual change we have called the transition from ELSA 1 to ELSA 2.

It is pointless to attempt to capture ELSI by simple definitions and descriptions, positive or negative. In this sense, the researchers have never been ELSI researchers – there is no such thing. However, the development and discussions within this interdisciplinary field have created a set of ELSI capacities that hinges on a diversity of approaches. We will therefore suggest that the ELSI field incorporates three wide areas of expertise that all contribute to knowledge building within the field. We will furthermore suggest that these expertise areas through interactions with the technosciences and with each other are gradually transformed in ways that result in new methods and theories. These three groups build their knowledge on scholarly achievements within the humanities, social sciences as well as the natural sciences themselves.

### Three approaches, three disciplines together creating pluralism within ELSI

There are at least three main groups of researchers or perspectives operating in the interdisciplinary ELSI research field: Ethicists, social researchers and scientists/technologists/practitioners. We have made this classification based on the project catalogue of the former ELSA programmes of the Research Council of Norway, as well as other research programmes. The basic training and education background largely determines the subject matter, methodology and analyses of their research.

The characteristics of the three approaches are respectively:

1. Normative analysis typically developed through conceptual analysis by ethicists and epistemologists with training in philosophy, theology or law.
2. Studies of stakeholder opinion or studies of how scientific and social activities mutually interact typically undertaken by scholars trained in STS and/or fields like sociology, anthropology and history
3. Scientific analysis of technical issues like risk research, typically developed by researchers with a science and technology background.

The first group was arguably the dominant one in the early days of ELSA studies, and much of the criticism we have discussed above target this kind of research. Traditionally, they focus on the normative consequences of technology developments or discuss ethical issues as they arise in concrete cases. This kind of applied ethics is valuable not only as a way to understand the empirical issues at stake; it also serves as an interpretation of the theory in a new setting (Gadamer 1990). This approach certainly risks degenerating into scholastic discussions of farfetched technological futures, defending spectacular positions or providing the technology proponents with the answers they seek. However, the mere diversity of normative ELSA literature gives little support to these as being dominant varieties. Rather, applied ethics research has provided important and diverse arguments directly relevant for policy discussions. Leading researchers from this part of ELSA research has taken central roles in governmental advisory boards and in the best cases contributed to a wider and better-informed public debate.

The second group of ELSA researchers initially continued the kind of research they already engaged in within other fields, using methodology from social sciences in order to understand how the users or those affected by specific phenomena perceived them, such as the European public scepticism towards the use of biotechnology in food production (e.g. Gaskell et al. 2003). One important outcome of this research was an increased awareness of the significance of public engagement in technology regulation (Stirling 2008). In this way, the social science research served an important role as an instigator of normative changes in science politics and regulation. Simultaneously, the social sciences studied the activities and practices of science and technology, enhancing our understanding of these activities as crucial and paradigmatic forms of modern life. One could say that these studies led to theoretical reflections analogous to the ones seen in applied ethics, and this continuous reflexive work has resulted in altered ways of doing social science studies of science and technology. It is important to note that despite the descriptive nature of social sciences, they served a very important normative role. This normative role has not been clearly acknowledged within the field, although there has been growing awareness of this role in later years.

ELSA research is interdisciplinary, and in some instances, discussing cases or general developments requires scientific and technological knowledge. The third group of ELSA researchers provide detailed technical knowledge and understanding of natural phenomena, particular technologies or professional practices due to their proper professional training within the field. Medical ethics is paradigmatic of ELSA research, as there have always been physicians actively participating in this kind of research. In many cases, these partial insiders are the ones who have raised the crucial questions. Also within other technology areas, such as agricultural biotechnology and the emerging fields of nanotechnology, scientists and technologists participate in ELSA research. They often go beyond established fields of risk assessments into more encompassing ethical-political issues on how to deal with scientific controversy and uncertainties. Their strengths are to large extents related to their understanding of the technical and scientific issues involved and grasp of concrete cases, their familiarity with laboratory work, as well as their credibility when communicating with fellow scientists. Thus both their approach and the basis for their credibility differs from that of the two other groups.

These three approaches are indispensable and complementary aspects of the ELSA field. We should be aware that even if their basic training, perspective and methodology are very different, there is increasing cooperation and exchange of theories and methods between the different groups. Although surveys and qualitative methods such as focus groups and consensus conferences, as well as other public engagement exercises come from social science methodology, researchers with philosophy or science backgrounds increasingly use these methods (e.g. Burgess and Tansey 2006). Representatives of all three groups exercise the new kinds of collaboration with science and technology research groups we associate with ELSA 2. Likewise, there is an increasing overlap in theoretical literature. Deliberative democracy theories are parts of political philosophy as well as social sciences, and all three groups may refer to post-normal science or related frameworks, when discussing risk and uncertainty or public engagement. Even sociologists may discuss normative questions referring to literature from the philosophical ethics canon.

Despite these converging tendencies, the three groups remain distinct, and we will argue this is beneficial for several reasons. The subject matter of ELSA research is diverse, and will need different kind of competences, theoretical approaches and methodologies in order to meet the different challenges. Action research type of research integrated in ongoing technology projects is merely one answer to the challenges raised within the field. In other cases one may better perceive what is at stake by analysing the technology development from the outside, utilising minority studies perspectives or Kantian ethics to mention but two. Another reason is that as long as ELSA is a field of interdisciplinary cooperation, solid disciplinary competence is necessary. There are researchers with “double competence” but they are and will be the exceptions. There is no doubt that knowledge production in the ELSA field requires proper training within well-established disciplines.

## Conclusion

Articulating values is always more than a purely descriptive project, as the theoretical frameworks we employ in studying human action invariably contain some notion of the good (Taylor 1985). So describing the values involved in technological practices from different theoretical perspectives, means evaluating the good of these practices from different viewpoints. As the different approaches not only bring to light different aspects of technological practices, but do this based on different sets of implicit value judgements, we hold that an adequate approach to ELSA studies should strive to keep alive pluralism in professional training, theoretical frameworks, methodologies and perspectives.

This is not to say that every theory, method or perspective is equally adequate or provides good analyses in all cases. It is important that we use peer review processes and keep alive critical discussions in order to satisfy scientific quality requirements. Clearly, in a fairly novel and interdisciplinary research field, scientific standards are not fully established. It is plausible that lack of clear quality standards leads to disagreements concerning what one considers good research, and therefore it is important to ensure an inclusive approach combined with an ongoing open debate on standards for ELSA research.

The post-ELSI call suggests there exists a particular activity – ELSI research – that we ought to replace with something new, due to its shortcomings. We argue rather that the ELSI field consists of several disciplines working with a number of issues connected to new technology developments, employing a wide range of theories and methods, including those proposed in the post-ELSI manifesto. We have described how the field has been marked by disciplinary and methodological pluralism from the beginning, and that a significant shift to inclusion of engagement-oriented research took place several years ago. In fact, this valuable addition to the range of ELSI approaches may prove too successful. Norway and EU direct increasing proportions of research funding towards integrated research within large-scale technology project, which means less room for other methodologies and topics. This is the kind of research advocated in the manifesto.

The call for post-ELSI, be it successful, may similarly result in a narrowing down of the range of research activity within this area. Seen in this perspective, the manifesto serves particular research political interests, supporting increased funding for a particular branch of research within the interdisciplinary field of research, based on crude characteristics of ELSI insufficiently supported by normative analysis of fallacies of ELSI. There is no such thing as ELSI research. There are only ethicists, sociologists, STS scholars, anthropologists, physicians, biochemists, biotechnologists and a many others working side-by-side or together on ELSI issues. This plurality stands in no need of replacement.

## Endnotes

^a^Due to our focus on the ‘post-ELSI’ term we generally stick with the American ELSI acronym, which originally meant ‘Ethical, Legal and Social Implications’ and later altered to ‘Issues’. The latter is usually taken to be synonymous with the European ELSA: Ethical, Legal and Social Aspects. The scientific fields of which this research is studying the implications, issues or aspects, has expanded over time and in turn influenced the developments of ELSI research, as we will see. When we refer to the Norwegian or European research contexts, we use the ELSA acronym as this has been dominant there, both to designate the field and for the funding programs.

^b^There is an interesting contrast between the manifesto’s complaints of ELSI researchers as “nay-sayers” and Winner’s statement here. They cannot both be true at the same time.

^c^For the record, all three authors of this article did receive research funding through several of these calls so we have no personal axes to grind.
